# Bioinformatics analysis of microRNAs related to blood stasis syndrome in diabetes mellitus patients

**DOI:** 10.1042/BSR20171208

**Published:** 2018-03-21

**Authors:** Ruixue Chen, Minghao Chen, Ya Xiao, Qiuer Liang, Yunfei Cai, Liguo Chen, Meixia Fang

**Affiliations:** 1 School of Chinese Medicine, Jinan University, Guangzhou, Guangdong 510632, China; 2 Reproductive Center, Guangdong Women and Children Hospital, Guangzhou, Guangdong 511400, China; 3 Institute of Laboratory Animals, Jinan University, Guangzhou, Guangdong 510632, China

**Keywords:** bioinformatics, blood stasis syndrome, diabetes, endothelial cells, microRNA

## Abstract

In traditional Chinese medicine (TCM), blood stasis syndrome (BSS) is mainly manifested by the increase of blood viscosity, platelet adhesion rate and aggregation, and the change of microcirculation, resulting in vascular endothelial injury. It is an important factor in the development of diabetes mellitus (DM). The aim of the present study was to screen out the potential candidate microRNAs (miRNAs) in DM patients with BSS by high-throughput sequencing (HTS) and bioinformatics analysis. Human umbilical vein endothelial cells (HUVECs) were incubated with 10% human serum to establish models of DM with BSS, DM without BSS (NBS), and normal control (NC). Total RNA of each sample was extracted and sequenced by the Hiseq2000 platform. Differentially expressed miRNAs (DE-miRNAs) were screened between samples and compared with known changes in mRNA abundance. Target genes of miRNAs were predicted by softwares. Gene Ontology (GO) and pathway enrichment analysis of the target genes were conducted. According to the significantly enriched GO annotations and pathways (*P*-value ≤ 0.001), we selected the key miRNAs of DM with BSS. It showed that the number of DE-miRNAs in BSS was 32 compared with non-blood stasis syndrome (NBS) and NC. The potential candidate miRNAs were chosen from GO annotations in which target genes were significantly enriched (−log_10_ (*P*-value) > 5), which included miR-140-5p, miR-210, miR-362-5p, miR-590-3p, and miR-671-3p. The present study screened out the potential candidate miRNAs in DM patients with BSS by HTS and bioinformatics analysis. The miRNAs will be helpful to provide valuable suggestions on clinical studies of DM with BSS at the gene level.

## Introduction

Diabetes mellitus (DM) is a chronic disease caused by lack of insulin secretion or lower biological effects of insulin. It is characterized by metabolic disorders of glucose, protein, and fat metabolism. The International Diabetes Federation (IDF) reported that 8.4% of all-cause deaths were attributable to DM in adults aged 20–79 years globally in 2014 [1]. It reported that there were 415 million patients with DM around the world in 2015, and there will be 642 million DM patients estimated in 2040. It also reported that every 6 s a person dies from diabetes and 1 in 7 births are affected by gestational diabetes [2]. DM patients often have insulin resistance (IR) or compensatory insulin, which can cause organs involvement and finally lead to diseases, such as coronary heart disease (CHD), obesity, hypertension and dyslipidemia.

Modern pathological studies have shown that the vascular endothelium is damaged by high blood sugar in the early stage of diabetes, which leads to slow blood flow and ischemia, which is the main cause of death and disability in DM patients [3]. Blood stasis is considered to be a basic pathogenic factor of DM in traditional Chinese Medicine (TCM) and occurs in many diseases, such as hypertension, CHD, DM, metabolic diseases, etc. [4–7]. Blood stasis syndrome (BSS) has been studied in recent years, including in hemorheology, microcirculation, platelet function, vascular endothelial function, and molecular biology. Studies on the mechanism of BSS have been limited in blood, but have focused on the relationship between vascular changes and the occurrence of BSS. Blood stasis biology has been associated with changes in vascular endothelial cell function; moreover, endothelial cell damage has been proved closely associated with the development of BSS [8,9].

BSS, which blocks the heart and vessels, not only refers to blood stagnation or coagulation in blood vessels but also includes the permeation of blood into perivascular tissues, skin, etc. Pathogenesis of BSS is very complicated, involving multiple systems, and a variety of tissues [10]. For example, BSS is the main pathogenesis of CHD. Modern pharmacological studies showed that activating blood stasis therapy could effectively improve the etiology and pathogenesis of CHD [11–14]. According to the differences in the internal and external environment of the individual disease, BSS were divided into qi-deficiency and blood stasis syndrome (QDBS), qi-stagnation and blood stasis syndrome (QSBS), cold-coagulation and blood stasis syndrome (CCBS), heat-accumulation and blood stasis syndrome (HABS). However, limited data are available on the mechanism of BSS.

miRNAs are a type of short, non-coding RNA molecule which can inhibit effective mRNA translation of the target genes via imperfect base pairing or lead to mRNA degradation via complementary base pairing [15]. Over 2500 miRNA have been discovered in humans (miRBase database www.mirbase.org), and many of them are found to be linked to various kinds of diseases. Thus, miRNA can be used as biomarkers, clinical diagnostic and therapeutic targets, such as miR-122 and miR-199a [16]. In our previous studies, we analyzed miRNAs and mRNAs levels in endothelial cells exposed to serum from hypertensive patients with BSS and found some potential candidate miRNAs and gene targets [17,18].

miRNAs play key roles in the pathological process of DM. They can regulate the development of pancreatic cells, the release of insulin and the function to peripheral target tissues, finally regulating glucose metabolism [19–21]. For example, miR-375 regulates genes controlling cell growth and proliferation, which is essential for normal glucose homeostasis, α- and β-cell turnover, and adaptive β-cell expansion in response to increasing insulin demand in IR [20]. The relationship between miRNAs and DM has been studied more, but there are few reports about the relationship between miRNAs and BSS in DM. Therefore, there is need to study miRNAs related to BSS in DM patients, to provide valuable suggestions on clinical diagnosis and treatment of DM with BSS. In the present study, DM patients were diagnosed as different TCM syndromes according to different symptoms, and were divided into QDBS, QSBS, CCBS, HABS, and NBS. Hyperglycemia and blood stasis in diabetic patients are an important factor in vascular endothelium damage. So we constructed four kinds of BSS diabetic endothelial cell models, and analyzed the regulatory mechanism of BSS-associated miRNAs by high-throughput sequencing (HTS) technology, compared with non-blood stasis diabetes and normal endothelial cells.

## Materials and methods

### Patients

Patients were diagnosed according to the 2010 Chinese Medical Association ‘Type 2 diabetes prevention guidelines in China’ and the Chinese Association of the Integration of Traditional and Western medicine ‘Consensus of Integrative Medicine on BSS Diagnosis and Treatment in 2011’ [22,23]. Patients were enrolled from the first affiliated hospital of Jinan University (Guangzhou, CHN). A total of 40 cases of DM patients with BSS were divided into QDBS (*n* = 10), QSBS (*n* = 10), CCBS (*n* = 10), and HABS (*n* = 10). There were also 40 cases of DM patients without BSS (NBS).

Diagnostic criteria of BSS were meeting two or more than two of the following criteria. (I) Dark purple or dark red tongue, or with petechia or ecchymosis. (II) Bluish purple or dark color of the complexion, lip, gingiva, periocular skin, finger tip (or toe-end). (Ⅲ) Scaly dry sin, varicosity or abnormal telangiectasia in different parts of the body. (IV) Constant pain, pricking, or colic pain. (V) Stagnant blood or hematocele (in viscera, tissue, serous cavity, or under the skin due to bleeding), or intermittent claudication. (VI) Amenorrhea or dark menstruation with blood clot. (VII) Numbness of limbs or hemiplegia. (VIII) Psychomania or amnesia. (IX) Hesitant pulse, or knotted and intermittent pulse, or asphygmia. (X) Abdominal pain with resistance of pressing. (XI) Viscera enlargement, neoplasm, inflammatory or non-inflammatory lump, hyperblastosis. Patients with interventional therapy or surgical operation who do not meet this criterion should be excluded.

The typical symptoms of QDBS, QSBS, CCBS, and HABS were as follows. QDBS: Physically and mentally fatigued, pant, sweating, dark and big tongue with thin and white fur and tooth marks, knotted and intermittent pulse. QSBS: Distension, depression or pain in the chest, hypochondrium or stomach, irritability, dark tongue, stringy pulse. CCBS: Intolerance to cold and cold limbs, peripheral coldness, exacerbated by exposure to cold, pale face, pale tongue with white fur, deep pulse, tight pulse, slow pulse, or taut pulse. HABS: Fever, ozostomia, bitter taste, xerostomia, astriction or yellow urine, dark red tongue with yellow thick fur, rapid pulse, or slippery pulse.

The characteristics of DM patients with BSS compared with NBS were as follows: The average random blood glucose (14.12 ± 1.28 in BSS vs. 11.81 ± 2.40 in NBS) mmol/l, fasting blood glucose (7.95 ± 0.84 in BSS vs. 8.97 ± 1.74 in NBS) mmol/l, oral glucose tolerance test (OGTT) and 2 h blood glucose (13.06 ± 1.31 in BSS vs. 11.86 ± 1.23 in NBS) mmol/l. There was no significant difference in the random blood glucose, fasting blood glucose, and OGTT 2 h blood glucose (*P* > 0.05). Thirty healthy volunteers were recruited from Jinan University as normal controls (NC). Fasting venous blood and self-coagulation samples were centrifuged (4°C, 2000 rev/min, 15 min), and the supernatants were transposed into a sterilized EP tube, and then incubated in a water bath at 56°C for 30 min to inactivate serum complement. Finally, the samples were stored at −20°C. All samples were equally mixed in each group before use. Potential participants that were interested in the present study received a complete explanation of the protocol and signed the consent form. The ethical approval for the study was permitted by the Ethics Committee of the Medical College of Jinan University.

### Cell models

CRL-1730 HUVECs (ATCC, USA) were cultured in culture flasks (1 × 10^5^/ml, 25 ml) in Dulbecco’s modified Eagle’s medium (DMEM) (Gibco, USA) containing 10% fetal bovine serum (FBS) (Gibco, USA) for 24 h at 37°C plus 5% CO_2_. The supernatant was discarded and the cells were washed with phosphate-buffered saline (PBS) (Gibco, USA) and then cultured with serum-free DMEM for 24 h. The supernatant was then discarded and the cells were washed with PBS. Then a 10% serum of QDBS, QSBS, CCBS, HABS, NBS, and NC was added to six culture flasks for 24 h. Finally, the cell models of QDBS, QSBS, CCBS, HABS, NBS, and NC were established. The supernatant was discarded and the cells were washed with PBS. The cells were digested in the culture flasks for 3 min with 1 ml TRIzol and the cells observed under an inverted microscope. Then, cells were collected into freezing tubes and stored at −80°C to preserve.

### Small RNA sequencing

Total RNA was extracted from cells by TRIzol (Invitrogen, USA). The small RNA library (140–160 bp) was constructed by Illumina Truseq Small RNA. The human small RNA sequence analysis was performed by the Hiseq2000 platform with 1 * 51 bp. Original images were transformed into raw reads by Base Calling. Quality control of the reads was conducted by SeqPrep (https://github.com/jstjohn/SeqPrep), Sickle (https://github.com/najoshi/sickle), and Fastx-Toolkit (http://hannonlab.Cshl.edu/fastx_toolkit/). The clean reads should satisfy the following criteria. (I) No reads with adaptors. (II) No reads with more than 10% unknown bases. (III) No low-quality reads. (IV) The length of the reads should be 18–32 bp. Then, further analysis can be conducted. The sequences of the same reads were combined to obtain the unique sequence, which was used to analyze the species and abundance of small RNAs in the statistical samples. The annotation of measured small RNAs was mapped to Rfam database (http://Rfam.sanger.ac.uk/) and GenBank noncoding RNA database (http://blast.ncbi.nlm.nih.gov/), thus filtering out the rRNA, scRNA, snoRNA, snRNA, tRNA, and other non-coding RNA. The remaining sRNAs were mapped to the human miRNA data of miRBase 21.0 (http://mirbase.org) to identify the known miRNAs. And the novel miRNAs were predicted by miRDeep2 (https://www.mdc-berlin.de/8551903/en/research/research_teams/systems_biology_of_gene_regulatory_elements/projects/miRDeep), randfold (http://www.bits.vib.be/index.php?option=com_content&view=article&id=16382011:randfold&catid=15:sequence-based&Itemid=636), Bowtie (http://bowtie-bio.sourceforge.net/index.shtml), and RNAfold (http://www.tbi.univie.ac.at/RNA/).

### MicroRNA screening

In order to get DE-miRNAs, on the basis of miRNA expression profiles, four comparisons were made, including QDBS vs. NBS and NC, QSBS vs. NBS and NC, CCBS vs. NBS and NC, HABS vs. NBS and NC. We used TMM (trimmed means of *M*-values method) to standardize read count data obtained in the analysis of miRNA expression level, then applied DEGseq to do the difference analysis using a Benjamini *q*-value of 0.001 as the cut-off (corrected *P*-value < 0.001), were used to screen out the DE-miRNAs [24]. Then the common DE-miRNAs in the four comparisons were selected for the following target prediction by Microsoft Excel software.

### Bioinformatics analysis of miRNA

The two softwares microRNA.org (http://www.microrna.org/) and TARGETSCAN (http://www.targetscan.org/) were utilized for predicted target genes of the DE-miRNAs in BSS. Importantly, the common predicted genes of the software were selected for the following bioinformatics analysis. And, target genes predicted with the cumulative weighted context ++ score less than −0.2, were selected for further analysis. To better identify the miRNAs important for regulating BSS, the predictions for miRNA target genes were compared with the differentially expressed mRNA transcripts (DE-mRNAs; fold change > 2; *P*-value < 0.05) identified from an analysis of the whole transcriptomes determined for these samples (unpublished data). Examples showing a negative correlation between the changes in abundance of a DE-miRNA with its predicted DE-mRNA partner were retained for further analysis. GO (Gene Ontology) and pathway enrichment analysis were used to infer the functional annotations of the genes for DM with BSS by MAS (Molecule Annotation System, http://bioinfo.capitalbio.com/mas3/), which is a web-based software toolkit for whole data mining and function annotation solution to extract and analyze biological molecule relationships from the public knowledgebase of biological molecules and signification, including GenBank, EMBL, SwissProt, Gene Ontology, KEGG, BioCarta, GenMapp, mirBase, EPD, HPRD, MIND, BIND, Intact, TRANSFAC, UniGene, dbSNP, OMIM, InterPro, HUGO, MGI, RGD, etc. In addition, the significantly enriched GO annotations were chosen (−log_10_(*P*-value) > 5). The predicted targets of miRNAs in BSS were classified according to pathway analysis. The target genes were chosen from the significantly enriched GO annotations. Their miRNAs were selected as the potential candidate miRNAs of BSS in DM.

### Online data deposition

The data discussed in this publication have been deposited in NCBI Sequence Read Archive and is accessible through GEO Series accession no. GSE109265 (the transcriptome data) (https://www.ncbi.nlm.nih.gov/geo/query/acc.cgi?acc=GSE109265) and no. GSE109266 (the miRNA data) (https://www.ncbi.nlm.nih.gov/geo/query/acc.cgi?acc=GSE109266).

## Results

### Sequencing data quality

**Figure 1:**
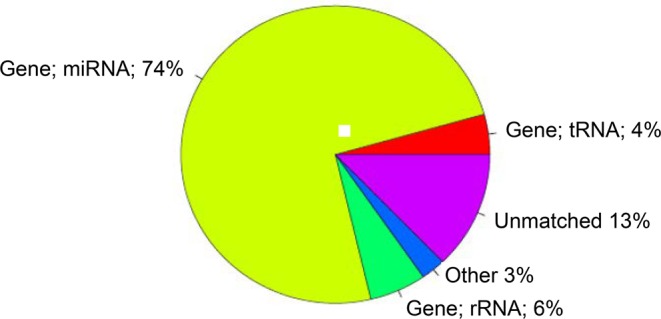
Proportion of small RNAs reads by Rfam database.

Clean reads of small RNAs were filtered from raw reads after quality control. The unique clean reads were annotated by Rfam and non-miRNA reads, such as rRNA, scRNA, snoRNA, snRNA, and tRNA were abandoned. The proportion of miRNA reads of all samples by the Rfam database accounted for 74% (Figure 1). The clean miRNA reads of each sample were mapped to the human reference genome. The results of sequence statistics among the samples and miRNA read numbers of all samples are listed in Table 1.

**Table 1:** Summary of sequence statistics of the samples

Group	Total reads	High quality	Clean reads	Mapped clean reads	Unique reads
QDBS	26359269	12893670	11531953	10038161	944791
QSBS	28406778	8737699	8255960	6778666	787743
CCBS	30872981	13804320	12959680	10723503	835698
HABS	29157502	10311967	9753552	7973897	801745
NBS	40339424	11231087	10768288	9211771	590405
NC	17440198	15261312	6297952	3815519	253955

### Differentially expressed miRNAs between samples

The DE-miRNAs between groups were analyzed. Compared with NBS, the number of DE-miRNAs was as follows, QDBS (*n* = 359, 138 down-regulated, 221 up-regulated), QSBS (*n* = 394, 229 down-regulated, 165 up-regulated), CCBS (*n* = 372, 136 down-regulated, 236 up-regulated), and HABS (*n* = 346, 168 down-regulated, 178 up-regulated). Compared with NC, the number of DE-miRNAs was as follows: QDBS (*n* = 696, 270 down-regulated, 426 up-regulated), QSBS (*n* = 745, 331 down-regulated, 414 up-regulated), CCBS (*n* = 731, 278 down-regulated, 453 up-regulated), and HABS (*n* = 766, 334 down-regulated, 432 up-regulated).

### Differentially expressed miRNA in BSS

The common DE-miRNAs in QDBS, QSBS, CCBS, and HABS were identified as the differentially expressed miRNAs of BSS. The number of DE-miRNAs in BSS was 105 (59 down-regulated, 46 up-regulated) compared with NBS, while the number was 358 (143 down-regulated, 215 up-regulated) compared with NC, and the number was 32 (15 down-regulated, 17 up-regulated) compared with NBS and NC (Table 2).

**Table 2:** The DE-miRNAs in BSS, log_2_ (FC)

miRNA	BSS vs. NBS (NC)			
	QDBS	QSBS	CCBS	HABS
**Up-regulated**				
hsa-miR-1292-3p	4.69 (7.01)	5.76 (8.07)	5.92 (8.24)	4.71 (7.03)
hsa-miR-140-5p	0.28 (2.76)	0.18 (2.66)	0.3 (2.78)	0.14 (2.63)
hsa-miR-155-5p	0.28 (4.48)	0.41 (4.6)	0.23 (4.42)	0.4 (4.6)
hsa-miR-18a-5p	0.12 (2.8)	0.13 (2.81)	0.27 (2.95)	0.11 (2.79)
hsa-miR-20a-3p	0.83 (3.63)	0.59 (3.38)	0.57 (3.36)	0.51 (3.31)
hsa-miR-221-3p	0.17 (2.48)	0.75 (3.06)	0.62 (2.93)	0.15 (2.46)
hsa-miR-23a-3p	0.54 (3.02)	0.57 (3.05)	0.35 (2.83)	0.25 (2.73)
hsa-miR-362-5p	0.3 (2.96)	0.52 (3.18)	0.35 (3.01)	0.17 (2.82)
hsa-miR-3679-5p	1.62 (10.38)	1.43 (10.19)	0.9 (9.66)	1.44 (10.2)
hsa-miR-374a-5p	0.43 (6.45)	0.64 (6.65)	0.81 (6.83)	0.25 (6.27)
hsa-miR-374b-5p	0.6 (3.51)	1.02 (3.93)	0.61 (3.52)	0.39 (3.31)
hsa-miR-4423-3p	2.3 (8.88)	2.49 (9.07)	1.66 (8.24)	2.44 (9.03)
hsa-miR-4517	1.45 (4.59)	0.56 (3.7)	1.23 (4.37)	0.86 (4)
hsa-miR-4521	0.83 (7.22)	0.15 (6.54)	0.54 (6.93)	0.43 (6.82)
hsa-miR-590-3p	0.81 (2.89)	1.17 (3.24)	1.05 (3.12)	0.71 (2.79)
hsa-miR-7-1-3p	0.79 (2.53)	0.41 (2.16)	0.31 (2.05)	0.37 (2.12)
hsa-miR-96-5p	0.41 (5.14)	0.14 (4.87)	0.53 (5.26)	0.25 (4.98)
**Down-regulated**				
hsa-miR-1307-5p	−1.13 (−3.92)	−1.17 (−3.96)	−0.9 (−3.7)	−0.45 (−3.24)
hsa-miR-130b-3p	−0.39 (−0.35)	−0.14 (−0.1)	−0.6 (−0.56)	−0.2 (−0.16)
hsa-miR-151a-3p	−0.33 (−0.3)	−0.15 (−0.12)	−0.2 (−0.25)	−0.16 (−0.14)
hsa-miR-181c-3p	−0.59 (−1.21)	−0.44 (−1.06)	−0.25 (−0.87)	−0.59 (−1.21)
hsa-miR-192-5p	−0.38 (−2.75)	−0.34 (−2.71)	−0.16 (−2.53)	−0.15 (−2.51)
hsa-miR-196a-3p	−1.06 (−0.97)	−0.68 (−0.59)	−0.87 (−0.78)	−0.54 (−0.45)
hsa-miR-210	−0.54 (−0.3)	−0.81 (−0.57)	−0.77 (−0.53)	−0.42 (−0.18)
hsa-miR-22-3p	−0.32 (−1.03)	−0.01 (−0.73)	-0.12 (−0.84)	−0.13 (−0.84)
hsa-miR-29b-3p	−0.71 (−0.15)	−0.65 (−0.09)	−0.67 (−0.11)	−0.64 (−0.08)
hsa-miR-29c-3p	−0.44 (−4.71)	−0.53 (−4.8)	−0.31 (−4.58)	−0.38 (−4.65)
hsa-miR-30a-5p	−0.09 (−1.24)	−0.25 (−1.4)	−0.22 (−1.37)	−0.04 (−1.19)
hsa-miR-30e-5p	−0.44 (−3.01)	−0.48 (−3.05)	−0.35 (−2.92)	−0.27 (−2.84)
hsa-miR-378c	−0.8 (−1.44)	−0.44 (−1.07)	−0.69 (−1.33)	−0.51 (−1.14)
hsa-miR-378d	−0.79 (−1.38)	−0.56 (−1.14)	−0.79 (−1.37)	−0.73 (−1.31)
hsa-miR-671-3p	−0.51 (−1.86)	−0.4 (−1.74)	−0.89 (−2.23)	−0.6 (−1.95)

### GO and pathway analysis

**Figure 2:**
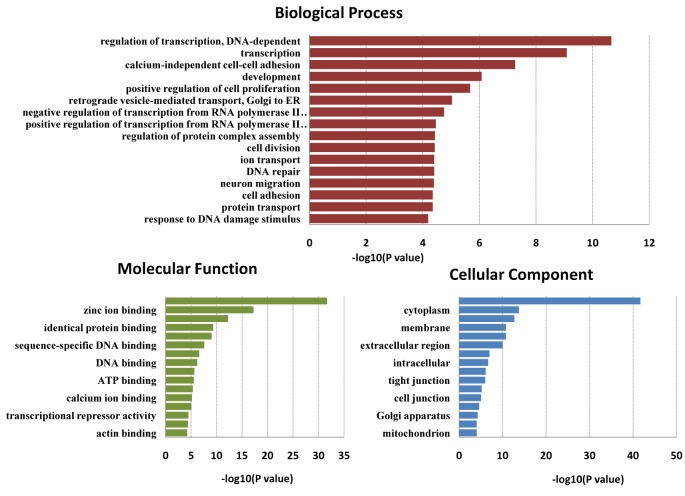
GO enrichment analysis of 111 DE-mRNAs in BSS, compared with NBS and NC.

**Figure 3:**
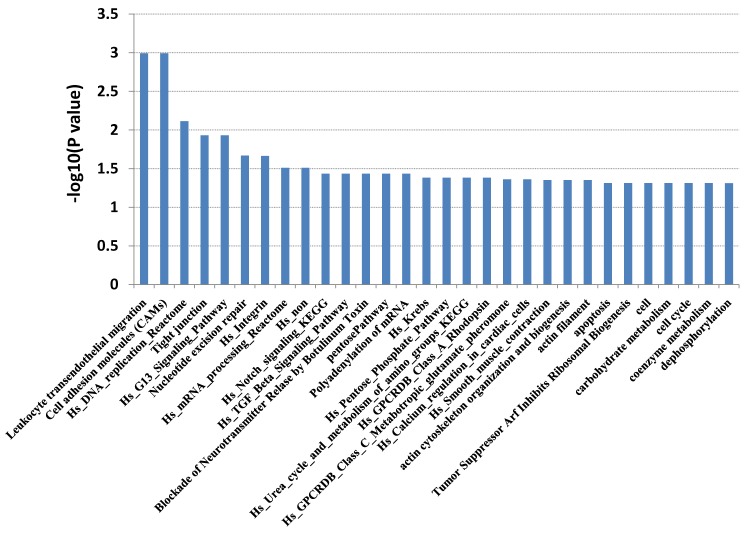
Pathway enrichment analysis of 111 DE-mRNAs in BSS, compared with NBS and NC.

Candidate target genes for the 32 DE-miRNAs in BSS were predicted bioinformatically and compared with mRNAs identified as being differentially expressed in the analysis of the transcriptomes for the same samples (unpublished data). Identification of the expected negative correlation between the expression of a miRNA and its candidate target gene produced a final list of 32 DE-miRNAs putatively targeting 111 DE-mRNAs. Then, all the genes were analyzed by GO and pathway analysis. GO analysis classified genes by biological process, molecular function and cellular component. In biological process, genes were mainly enriched in regulation of transcription (DNA-dependent). In molecular function, genes were mainly enriched in protein binding. In cellular component, genes were mainly enriched in nucleus. This revealed that most genes were involved in cellular process and physiological processes (Figure 2). Pathway analysis suggested that the genes primarily active in leukocyte transendothelial migration, cell adhesion molecules (CAMs), hs DNA replication reactome, tight junction, and hs G13 signaling pathway (Figure 3).

### The potential candidate miRNAs

There were 20 significantly enriched GO annotations that were chosen (−log_10_ (*P*-value) > 5). Twenty-four target genes of BSS were selected from the significantly enriched GO annotations in the 111 annotated genes (Table 3). Their miRNAs were selected as the potential candidate miRNAs of BSS in DM, which were hsa-miR-140-5p, hsa-miR-210, hsa-miR-362-5p, hsa-miR-590-3p, and hsa-miR-671-3p (Table 4).

**Table 3:** The significantly enriched GO annotations (-log_10_ (*P*-value) > 5)

No.	GO term	−log_10_ (*P-*value)	Protein
1	Nucleus	41.69	SLC2A4RG; PDX1; NIP7; UPF3A; ZNF655; RFC5; TBX2; ZNF576; CINP; JDP2; OSR2; ZNF282
2	Protein binding	31.67	PDZD4; ARFRP1; PDX1; NIP7; UPF3A; ZNF655; TIMM44; TBX2; CD81; MLPH
3	Zinc ion binding	17.24	SLC2A4RG; ZNF655; ZNF576; MLPH; OSR2; ZNF282
4	Cytoplasm	13.73	SLC2A4RG; UMODL1; PDZD4; IER2; UPF3A; RABEP2; MLPH
5	Cytoplasm	13.73	SLC2A4RG; UMODL1; PDZD4; IER2; UPF3A; RABEP2; MLPH
6	Integral to membrane	12.66	UMODL1
7	Metal ion binding	12.28	SLC2A4RG; ZNF655; ZNF576; MLPH; OSR2; ZNF282
8	Plasma membrane	10.74	UMODL1; GRM5
9	Membrane	10.74	TIMM44; CD81
10	Regulation of transcription, DNA-dependent	10.66	SLC2A4RG; ZNF655; ZNF576; JDP2; ZNF282
11	Extracellular region	10.00	UMODL1; NOG
12	Transcription	9.09	SLC2A4RG; ZNF655; ZNF576; JDP2; ZNF282
13	Transcription factor activity	9.04	SLC2A4RG; PDX1; TBX2; JDP2
14	Sequence-specific DNA binding	7.6	PDX1; TBX2; JDP2
15	Integral to plasma membrane	6.99	GRM5; CD81
16	Intracellular	6.67	SLC2A4RG; ARFRP1; ZNF655; ZNF576; CAPN6; OSR2; ZNF282
17	Nucleotide binding	6.6	ARFRP1; UPF3A; TIMM44;RFC5
18	DNA binding	6.19	ZNF655; ZNF576; ZNF282
19	Development	6.08	PDX1; TBX2
20	Positive regulation of cell proliferation	5.67	PDX1; TBX2; CD81; OSR2

**Table 4:** The potential candidate miRNAs in BSS

MiRNA	Target gene	Description
hsa-miR-140-5p	SLC2A4RG	SLC2A4 regulator
	UMODL1	Uromodulin-like 1
hsa-miR-210	PDZD4	PDZ domain containing 4
	ARFRP1	ADP-ribosylation factor related protein 1
	NSUN5	NOL1/NOP2/Sun domain family, member 5
	PDX1	Pancreatic and duodenal homeobox 1
	GRM5	Glutamate receptor, metabotropic 5
	UMODL1	Uromodulin-like 1
hsa-miR-362-5p	NIP7	Nuclear import 7 homolog (*Saccharomyces cerevisiae*)
	IER2	Immediate early response 2
	UPF3A	UPF3 regulator of nonsense transcripts homolog A
	ZNF655	Zinc finger protein 655
hsa-miR-590-3p	TIMM44	Translocase of inner mitochondrial membrane 44 homolog
	RFC5	Replication factor C (activator 1) 5, 36.5kDa
	TBX2	T-box 2
	ZNF576	Zinc finger protein 576
	SLC2A4RG	SLC2A4 regulator
	RABEP2	Rabaptin, RAB GTPase binding effector protein 2
hsa-miR-671-3p	CAPN6	Calpain 6
	CINP	Cyclin-dependent kinase 2-interacting protein
	CD81	CD81 molecule
	MLPH	Melanophilin
	JDP2	Jun dimerization protein 2
	OSR2	Odd-skipped related 2 (*Drosophila*)
	ZNF282	Zinc finger protein 282
	UMODL1	Uromodulin-like 1
	RABEP2	Rabaptin, RAB GTPase binding effector protein 2
	NOG	Noggin
	ZNF655	Zinc finger protein 655

## Conclusions and discussion

BSS is a syndrome relevant to TCM theory, which associated with many cardiovascular diseases, such as hypertension, diabetes mellitus. The lesion has a very obvious vascular endothelial injury. In study, four different types of BSS cell models were established and compared with NBS and NC. Blood stasis is common in these four comparisons. We obtained the miRNA related to BSS by screening their common miRNAs. Finally, it showed that the number of DE-miRNAs of BSS was 32 (15 down-regulated, 17 up-regulated) compared with NBS and NC. The potential candidate miRNAs were chosen from GO annotations in which target genes were significantly enriched (−log_10_(*P*-value) > 5), which included hsa-miR-140-5p, hsa-miR-210, hsa-miR-362-5p, hsa-miR-590-3p, and hsa-miR-671-3p.

Evidence for the relationship between miRNA and the vascular endothelium of DM was identified in modern studies. For example, experimental data have demonstrated that miR-126 can regulate vascularization by targeting Spred1 [25]. In addition, many miRNAs showed overexpression in DM rats, including miR-320, miR-291-5P, miR-129, etc., of which, miR-30 can down-regulate IGF1 (insulin-like growth factor 1) and its receptor [26]. Specifically, miR-320 and miR-21 can participate in ischemia-reperfusion injury by targeting corresponding genes [27,28].

Similar results were found in our study. Thirty-two miRNAs (15 down-regulated, 17 up-regulated) were found to be associated with BSS in DM by comparing four kinds of BSS with NBS and NC. GO analysis showed that their target genes were mainly associated with regulation of transcription, cell proliferation and division, calcium-independent cell-cell adhesion, cell adhesion, and protein binding. These are related to endothelial dysfunction and the expression of cell adhesion factors, which are associated with BSS [29–34]. The same results were found by pathway analysis. Their target genes were mainly enriched in leukocyte transendothelial migration, CAMs, and hs DNA replication reactome signaling pathway. The junctional adhesion molecule (JAM) family of proteins, JAM-B and JAM-C, are involved in polarized leukocyte transendothelial migration, and are expressed by vascular endothelial cells of the peripheral tissue and high endothelial venules in lymphoid organs. It was found that blockade with a neutralizing anti-JAM-C antibody can reduce the T1D incidence [35]. CAMs may be involved in the molecular mechanism of BSS, which are members of the immunoglobulin superfamily and are involved in synaptic rearrangements in the mature brain. Exenatide has been proved as a strong beneficial action in managing diabetes by altering gene expression of NCAM (neural cell adhesion molecule), ICAM (intercellular adhesion molecule), and VCAM (vascular cell adhesion molecule) [36]. Therefore, these miRNAs may be associated with diabetes secondary to BSS by promoting vascular injury and the expression of adhesion factors.

Based on the analysis, it showed that five miRNAs were closely related to BSS in DM, including hsa-miR-140-5p, hsa-miR-210, hsa-miR-362-5p, hsa-miR-590-3p, and hsa-miR-671-3p. Studies have proved these miRNAs are related to diabetes. The expression of miR-140-5p increases gradually with the increasing concentration of glucose in hyperglycemia-induced endothelial cells and was found to be involved in endothelial dysfunction [37]. A cross-sectional study, which sought to identify the profile of circulating miRNAs in T2D and its response to changes in insulin sensitivity, found a significant increase of miR-140-5p in T2D patients. Moreover, miR-140-5p was found to contribute independently to explain fasting glucose variance after controlling for confounders, and it could be down-regulated by metformin and insulin [38]. SLC2A4RG was predicted as a target gene of miR-140-5p and has been found closely related to DM. A study on pathway analysis of differentially regulated genes upon exercise revealed the regulators of SLC2A4RG was up-regulated in diabetic participants compared with NCs. The study provides novel insight into potential mechanisms to ameliorate the disturbed glucose and amino acid metabolism associated with T2D [39].

A study in Zucker diabetic fatty rats (ZDF rats) revealed that miR-210 was found increased over the course of the diabetic progression [40]. Another functional study indicated that modifications in the levels of miR-210 primarily resulted in increased β-cell apoptosis in diabetic mice [41]. miRNA PCR arrays in left ventricular specimens, which were collected from streptozotocin-induced diabetic mice, found a dysregulation of 316 out of 1008 total miRNAs compared with controls, and ingenuity pathway analysis revealed that miR-210 was implicated in myocardial signaling networks triggering oxidative stress [42]. As is known, the liver is a major organ in lipid metabolism and malfunctioning may lead to T2D. ARFRP1, which was predicted as the target gene of miR-210, has been proved to play an important role in lipoprotein maturation in the liver by influencing lipidation and assembly of proteins to the lipid particles [43]. Another target gene, PDX1, was identified as a key β-cell transcription factor, which is closely related to DM [44,45]. A study to evaluate miRNA involvement in gestational diabetes mellitus (GDM) discovered that miR-362-5p was significantly down-regulated in GDM, compared with normal controls [46]. Up-regulation of LDHA (lactate dehydrogenase A) is found in both human T2D and rodent T2D models. miR-590-3p can suppress LDHA and be used together with human embryonic stem cell (hESC) derived pancreatic endoderm (PE) transplantation into a high-fat diet induced T2D mouse model and significantly improved glucose metabolism and other symptoms of T2D [47]. Balanced deep-sea water (BDSW) was identified as a potential treatment for diabetes and obesity, which can enhance gene expression of TOMM40 and TIMM44 for mitochondrial protein import [48]. The current state of evidence for the relationship between miR-671-3p and DM has so far been unknown. However, its target gene CAPN6 (calpain 6) was identified to be responsible for diabetic nephropathy [49].

In summary, miRNAs can be used not only as biomarkers for the diagnosis of disease but also for natural regulation modes *in vivo*. In the present study, we established the endothelial cell model of DM with BSS, then screened out 32 potential miRNAs related to DM with BSS by HTS and bioinformatics. The five potential candidate miRNAs (hsa-miR-140-5p, hsa-miR-210, hsa-miR-362-5p, hsa-miR-590-3p, and hsa-miR-671-3p) may be important factors related to BSS in DM and can be used as biomarkers for diagnosis and drug targets for treating DM with BSS. However, there are some limitations in the present study, for example, the samples were pooled for sequencing with no replication. Therefore, further studies on the role of miRNAs and their putative target molecules identified in the present work will be required.

## Abbreviations

BSS: blood stasis syndrome
CAM: cell adhesion molecule
CHD: coronary heart disease
DM: diabetes mellitus
GO: Gene Ontology
HTS: high-throughput sequencing
HUVEC: human umbilical vein endothelial cell
IR: insulin resistance
JAM: junctional adhesion molecule
NBS: non-blood stasis syndrome
NC: normal control
OGTT: oral glucose tolerance test
PBS: phosphate-buffered saline
TCM: traditional Chinese Medicine
